# Comparison of complaints to the intensive care units and those to the general wards: an analysis using the Healthcare Complaint Analysis Tool in an academic medical center in Taiwan

**DOI:** 10.1186/s13054-018-2271-y

**Published:** 2018-12-06

**Authors:** Jih-Shuin Jerng, Szu-Fen Huang, Hsin-Yu Yu, Yi-Chun Chan, Huang-Ju Liang, Huey-Wen Liang, Jui-Sheng Sun

**Affiliations:** 1 Department of Internal Medicine, National Taiwan University Hospital, Taipei, Taiwan; 2 Department of Nursing, National Taiwan University Hospital, Taipei, Taiwan; 3 Center for Quality Management, National Taiwan University Hospital, Taipei, Taiwan; 4 Department of Physical Medicine & Rehabilitation, National Taiwan University Hospital, Taipei, Taiwan; 5 Department of Orthopedic Surgery, National Taiwan University Hospital, No. 7, Zhongshan South Road, Taipei, 100 Taiwan

**Keywords:** Intensive care unit, Healthcare complaints, Satisfaction, Patient-centered care, Quality of care, Patient safety

## Abstract

**Background:**

The management of complaints in the setting of intensive care may provide opportunities to understand patient and family experiences and needs. However, there are limited reports on the structured application of complaint analysis tools and comparisons between healthcare complaints in the critical care setting and other settings.

**Methods:**

From the complaint management database of a university-affiliated medical center in Taiwan, we retrospectively identified the records of healthcare complaints to the intensive care units (ICUs) from 2008 to 2016. Complaints to the general wards in the same period were randomly selected from the database with twice the number of that of the ICU complaints. We coded, typed, and compared the complaints from the two settings according to the Healthcare Complaint Analysis Tool.

**Results:**

We identified 343 complaints to the ICUs and randomly selected 686 complaints to the general wards during the 9-year study period. Most (94.7%) of the complaints to the ICUs came from the family members, whereas more complaints to the general wards came from the patients (44.2%). A total of 1529 problems (441 from ICU and 818 from general wards) were identified. Compared with the general ward complaints, in the ICU there were more complaints with multiple problems (25.1% vs. 16.9%, *p* = 0.002), complaints were referred more frequently to the nurses (28.1% vs. 17.5%, *p* < 0.001), and they focused more commonly on the care on the ICU/ward (60.5% vs. 54.2%, *p* = 0.029). The proportions of the three domains (clinical, management, and relationship) of complaints were similar between the ICU and general ward complaints (*p* = 0.121). However, in the management domain, the problems from ICU complaints focused more on the environment than on the institutional processes (90.9% vs. 74.5%, *p* < 0.001), whereas in the relationship domain, the problems focused more on communication (17.9% vs. 8.0%) and less on listening (34.6% vs. 46.5%) (*p* = 0.002) than the general ward complaints.

**Conclusions:**

A structured typing and systematic analysis of the healthcare complaints to the ICUs may provide valuable insights into the improvement of care quality, especially to the perceptions of the ICU environment and communications of the patients and their families.

**Electronic supplementary material:**

The online version of this article (10.1186/s13054-018-2271-y) contains supplementary material, which is available to authorized users.

## Background

Enhancing responsiveness to the expectations of the population is a fundamental goal of the healthcare system [1]. The healthcare institutions had sought to achieve this goal by understanding the satisfaction of the patients, as has been advocated in the critical care setting [2–5]. Information from the survey of satisfaction of intensive care unit (ICU) patients and families might infer issues for improvement of healthcare quality [6]. However, the information might be vague because respondents usually express a high level of satisfaction with the care [7] and the survey process may also deter them from expressing dissatisfaction due to concern about retribution by service providers [8, 9].

Recently, researchers and institutions have advocated the assessment of healthcare complaints as a complementary tool based on the views and perceptions of the patients and their families about the care and services. Healthcare complaints conventionally refer to an expression of grievance and dispute, typically written and communicated through a letter by a patient or their family, about the receipt of healthcare [10]. The complaints may request the correction of an ongoing problem or prevent its recurrence, while the complainers may believe they have valid information that the institution does not know or has failed to take sufficiently seriously. Similar to the evolution of surveys, the provision of complaints has also evolved to include multiple channels, such as e-mail and other electronic messaging services, and in-person contact with the patient relations office. This tool provides valuable information about people’s experience of the care and services received and their understanding of hospitals, patient safety, and healthcare quality [11, 12]. Since complaints are difficult to avoid in most clinical practice, reports recommend that the healthcare institutions include the analysis of complaints as a possible measurement of service quality [13–17]. Nevertheless, few institutions have established a structured analysis and management system for complaints, while most of the complaints are managed by patient relations or risk management system. This practice is in contrast to the fact that most institutes have established patient safety monitor systems such as incident reporting systems [12]. Moreover, there have been a limited number of studies about the complaints related to intensive care medicine [18]. An Australian study reported that the complaint rate in the ICU setting, an estimated 5.9 per 1000 patients, was similar to that in the general wards; the ICU complaints focused mainly on treatment, communication, and access [19]. However, in comparison with questionnaires on patient satisfaction, previous methods used to analyze ICU complaints were not well-standardized and were without a widely accepted framework. Recently, researchers have developed complaint coding taxonomy [11]. The Healthcare Complaint Analysis Tool (HCAT) advocated by Gillespie et al. [20] provides a reliable tool for coding complaints and measuring the severity of complaints, to facilitate service monitoring and organizational learning and assessment of healthcare complaints as an indicator of poor service outcomes [20].

As healthcare complaints to ICUs may reflect preventable, ongoing, important problems related to the care of patients treated in the ICU, analyzing these complaints may be beneficial for the healthcare providers and the organization, to prioritize the domains that need timely communication and improvements in care. Furthermore, analyzing ICU complaints may provide insights not easily drawn from other sources, although a structured approach is also required. In this study, we applied the HCAT to analyze and compare healthcare complaints to the ICUs and the general wards of an academic teaching medical center, to understand the types of complaints and to investigate the factors associated with the severity of problems raised by the complaints.

## Methods

We conducted this retrospective analysis at the National Taiwan University Hospital, Taipei, Taiwan. This study was approved by the institutional Research Ethics Committee C (#201706095 W) and was waived from the need for informed consent from the complainers or the patients.

The hospital, a 2300-bed, university-affiliated medical center with about 6400 full-time employees established the institutional Complaint Service Office (later renamed the Patient Relations Office) for management of healthcare complaints in 2008. The staff members of the office came from the Center for Quality Management of the hospital and served as the first-line correspondents for the complainers. They verified the complaint contents, collected pertinent information, contacted related workers of the hospital and the complainers and entered the data electronically into the institutional complaints management system. All of the verified complaints were filed and their contents were transcribed electronically into text files. The verbal contents from tape or video recordings of the complainers provided through non-written channels during the initial contact or in follow-up communications were also transcribed to text files. For this study, the investigators included all of the complaints to the ICUs during the study period and randomly selected twice the number of the complaints to the general wards during the same study period for comparison. The investigators retrieved relevant stored data from the office and coded the complaints through consensus for each complaint included in further analysis.

### Complaint coding

To obtain a quantitative description, the investigators (JSJ and HYY) coded each complaint according to the taxonomy from the Healthcare Complaints Analysis Tool (HCAT) [20]. Briefly, the coders independently analyzed the complaints by reviewing the three domains, including clinical, management, and relationship, and subdivided them into seven categories. The clinical domain pertains to quality of care and patient safety; the management domain refers to environmental management and institutional processes related to the handling of patients; the relationship domain includes communication, listening, and respect/patient rights [20]. After the problems were identified, the investigators clarified the related stages of care, the severity of the problems, and the degree of patient harms reported from the complaints. The coders then together reached a consensus for each case about the final coding results by discussing the available records related to the complaints. A detailed description of the process for applying HCAT in this study is provided in Additional file 1. Regardless of the original channels of the complaints, the coding of all of the complaints and problems in this study were exclusively based on the text records from the file database of the Patient Relations Office of this institution, while the severity of the complaint was assessed according to the contents of the text without any additional interviews with the staff members who originally managed the case or the healthcare workers caring for the patients.

### Additional information

We also determined the numbers of inpatients and inpatient days during the study period of 2008–2016, while the inpatient numbers and patient days in the ICUs were also calculated. We also looked at the institutional Incident Reporting System for information about whether had any incidents reported by the hospital employee to the hospital in relation to the complaints.

### Statistical analysis

We first analyzed the administrative characteristics related to the complaints and the types of complaints to the ICUs and the general wards derived by the HCAT. Then we compared the domains, categories, and types of problems identified from the complaints and the background characteristics such as complainer type, channels of complaint issuing, and staff groups to which the complaints referred. Multivariate analyses were performed to investigate the possible variables associated with an increased (medium or high) level of problem severity.

Results fom analysis of nominal variables are summarized and expressed as counts and percentages and age is expressed as median. Different categories, such as incident types and job types of the workers were compared using crosstabulations and the chi-square test. The Mann-Whitney U test was used to compare age in different groups of workers. A binary logistic regression model was applied to the analysis of variables associated with medium or high severity problems. Statistical analysis was performed using the SPSS 22 Software (SPSS Corp., Chicago, IL, USA). A *p* value <0.05 was considered statistically significant.

## Results

### Descriptive information about the complaints

During the 9-year study period, there were 21,886 healthcare complaints to the institution. These included 5866 (26.8%) complaints to the inpatient setting, 12,275 (56.1%) to the outpatient setting, 1494 (6.8%) to the emergency service setting and 2251 (10.3%) complaints with other or unspecified setting. From the complaints to the inpatient setting, we identified 343 (5.8%) eligible complaints to the ICUs. We then randomly selected 686 eligible cases of complaints to the general wards during the same study period as a comparison. During the study period, there were 735,134 inpatients, including 86,522 ICU admissions; these patients comprised 6,572,057 inpatient-days, including 637,774 patient-days in the ICUs and 5,934,283 patient days in the general wards. We then determined the rates of inpatient healthcare complaints as 8.0 cases per 1000 patients and 0.89 per 1000 patient-days. The complaint rates to the ICU were 4.0 cases per 1000 patients and 0.54 per 1000 patient-days, which were lower than the 8.5 cases per 1000 patients (*p* < 0.0001) and 0.93 per 1000 patient-days (*p* < 0.0001) for complaints to the general wards. The complaints to the ICUs included 130 (37.9%) to the medical ICUs, 77 (22.4%) to the surgical ICUs, and 136 (39.7%) to other types and mixed ICUs. During the same period, 25,693 patients were admitted to the medical ICUs and 34,884 to the surgical ICUs; therefore, the rate of complaints was higher for the medical than for the surgical ICUs (5.1 and 2.2 cases per 1000 patients, respectively, *p* < 0.0001).

Table 1 shows the comparison of administrative characteristics of the complaints. For the ICU complaints, 3.2% came from the patients, while most (94.7%) were from the family members; this was significantly different from the ward complaints that were more fequently from the patients themselves (49.1%), whereas only 44% were from the family members (*p* < 0.001). There was a greater proportion of complaints from individuals of unspecified gender to the ICU than to the general wards (39.1% vs. 19.5%, *p* < 0.001), but for the complainers with known gender, the ratios of female to male complainers were similar. The channels used for providing complaints were similar (*p* = 0.055), with opinion feedback sheets accounting for the major channel (59.8% and 58.7%, respectively). Several complaints were referred from complaints to the health authorities about the care in the hospital (*n* = 1 and *n* = 22 for the ICUs and general wards, respectively). By comparing the administrative data on complaints issued by the family and surrogates (327 to the ICUs and 304 to the general wards), we found that the ICU complaints were more often made using feedback sheets (59.6%) but fewer phone calls (19.6%) than ward complaints (42.8% and 30.96%) (*p* < 0.001). Among all of the complainers from the database with known gender (*n* = 761), the proportion of male complainers was similar among complaints to the ICU and the ward (43.5% vs. 42.6%, *p* = 0.810). However, among complaints issued by the family or surrogates (*n* = 453), there were more male complaints to the ICU than to the wards (43.1% vs. 33.6%, *p* = 0.038). Moreover, the ICU complaints were related to more issues with ongoing problems than complaints to the general wards (84.8% vs. 61.1%; *p* < 0.001) (Table 1).

**Table 1:** Cases of healthcare complaints related to intensive care units and the general wards

Variable	Complaints to the ICUs (*n* = 343)	Complaints to the general wards (*n* = 686)	*p* value
Source of the complaints			
Family member	326 (95.0%)	303 (44.2%)	< 0.001
Patient	11 (3.2%)	337 (49.1%)	
Other surrogate	1 (0.3%)	1 (0.1%)	
Hospital employee	1 (0.3%)	0 (0%)	
Visitor	0 (0%)	6 (0.9%)	
Unknown	4 (1.2%)	39 (5.7%)	
Gender of the complainer			
Male	91 (26.5%)	235 (34.3%)	< 0.001
Female	118 (34.4%)	317 (46.2%)	
N/A	134 (39.1%)	134 (19.5%)	
Complaint channel			
Opinion feedback sheet	205 (59.8%)	403 (58.7%)	0.055
Phone call	68 (19.8%)	148 (21.6%)	
In person	32 (9.3%)	55 (8.0%)	
E-mail	30 (8.7%)	42 (6.1%)	
Letter	5 (1.5%)	9 (1.3%)	
Fax	2 (0.6%)	7 (1.0%)	
Other	1 (0.3%)	22 (3.2%)	
Complained about ongoing problem			
Yes	291 (84.8%)	419 (61.1%)	< 0.001
No	52 (15.2%)	267 (38.9%)	

N/A: Not available from the complaint database

### Problems arising from the complaints, patient harms, and staff groups referred to

Of the 1029 complaints, we identified 1259 problems, including 441 in the complaints to the ICUs and 818 in the complaints to the general wards. There were more cases of complaints to the ICU with more than one issue per complaint than to the general wards (25.1% vs. 16.9%, *p* = 0.002). The average number of complaints per case was also higher in the ICU complaints than the ward complaints (1.29 and 1.19 for ICU and ward complaints, respectively, *p* = 0.002).

**Fig. 1:**
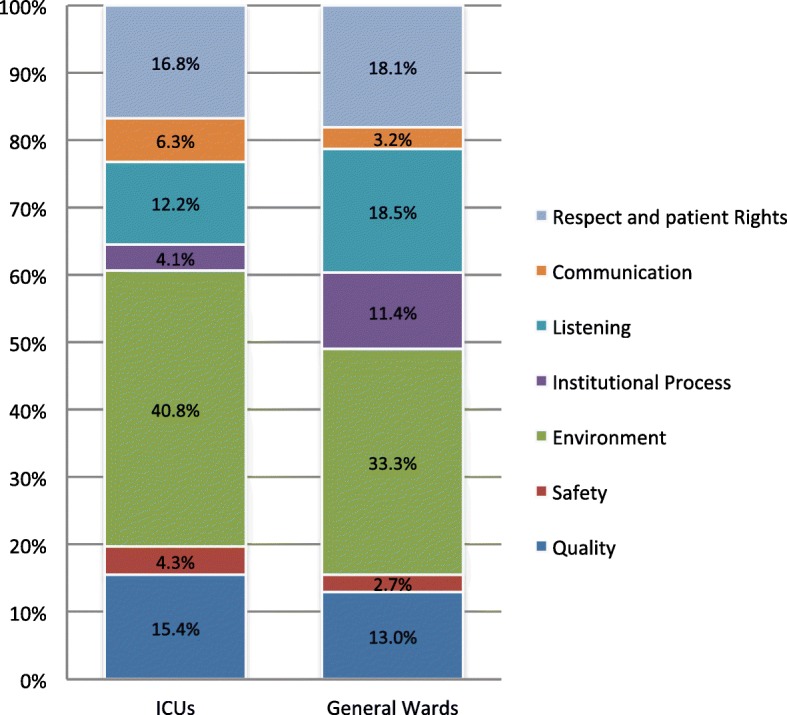
Proportions of the problem categories for the healthcare complaints to the ICUs and general wards

Table 2 summarizes the domains, categories, and types of problems identified from the healthcare complaints based on the classification from the HCAT, separating the ICU and ward complaints. The proportion of problems in the three domains (clinical, management, and relationship) was similar between the ICU and ward complaints (*p* = 0.121). While management problems were overall the most common domain in both complaint groups, the problems identified in the complaints issued by the family or surrogates were focused more on the relationship category (41.6%) than the complaints issued by the patients (32.8%) or other persons (30.0%) (*p* < 0.001). Problems identified in the complaints issued through the feedback sheets (*n* = 740) were significantly more focused on the management domain (56.6%) than the complaints issued through other channels (*p* < 0.001). Nevertheless, the proportions of problem domains were similar between ICU and ward complaints whether the complaints were issued through feedback sheet (*p* = 0.298) or other channels (*p* = 0.398). Figure 1 illustrates the proportions of seven categories of problems identified from the complaints to the ICUs and the general wards according to the suggestion by HCAT [20] (Fig. 1).

**Table 2:** Types of healthcare complaints related to intensive care units and the general wards

Problem domain	Problem category and type	Number of problems, ICUs (*n* = 441)	Number of problems, general wards (*n* = 818)	*p* value
Clinical		87	128	0.121
	Quality	68	106	0.394
	Bedding and dress	5 (7.4%)	5 (4.7%)	0.006
	Nutrition supply	1 (1.5%)	18 (17.0%)	
	Wound and indwelling device care	3 (4.4%)	3 (2.8%)	
	Patient handling	31 (45.6%)	25 (23.6%)	
	Patient monitoring	8 (11.8%)	13 (12.3%)	
	Patient involvement in care planning	7 (10.3%)	10 (9.4%)	
	Care outcome and sequel	13 (19.1%)	32 (30.2%)	
	Safety	19	22	
	Delay or mistake in diagnosis	0 (0%)	2 (9.1%)	0.498
	Medication event	1 (5.3%)	3 (13.6%)	
	Responding to change in clinical condition	8 (42.1%)	9 (40.9%)	
	Responding to clinical notification from the patient	5 (26.3%)	3 (13.6%)	
	Overlooking of clinical information	0 (0%)	1 (4.5%)	
	Teamwork and coordination problem	5 (26.3%)	4 (18.2%)	
Management		198	365	
	Environment	180	272	< 0.001
	Uncomfortable physical surroundings	68 (37.8%)	109 (40.1%)	0.002
	Accommodation problem	0 (0%)	5 (1.8%)	
	Ward cleanliness	5 (2.8%)	23 (8.5%)	
	Facility and equipment function	76 (42.2%)	92 (33.8%)	
	Staff availability and shortage	2 (1.1%)	14 (5.1%)	
	Interaction problem among patients and visitors	29 (16.1%)	29 (10.7%)	
	Institutional processes	18	93	
	Waiting for clinical visit or consultation	5 (27.8%)	38 (40.9%)	0.001
	Delay in medical procedure	6 (33.3%)	37 (39.8%)	
	Phone call or complaints not responded to	0 (0%)	1 (1.1%)	
	Appointment problem	0 (0%)	12 (12.9%)	
	Visiting time availability or scheduling	7 (38.9%)	4 (4.3%)	
	Maintenance of medical records	0 (0%)	1 (1.1%)	
Relationship		156	325	
	Listening	54	151	0.0016
	Staff ignorance of question and discomfort	2 (3.7%)	14 (9.3%)	0.055
	Dismissing of patient-provided information	4 (7.4%)	27 (17.9%)	
	Acknowledged problems not responded to, not addressed, or not followed through	48 (88.9%)	110 (72.8%)	
	Communication	28	26	
	Delayed communication of test results	1 (3.6%)	1 (3.8%)	0.303
	Incorrect or conflicted information given to the patient	3 (10.7%)	7 (26.9%)	
	Decision or plan not communicated	24 (85.7%)	18 (69.2%)	
	Respect and patient rights	74	148	
	Poor manner of the staff	50 (67.6%)	91 (61.5%)	0.702
	Not protecting private information	1 (1.4%)	3 (2.0%)	
	Staff self-control and patient discrimination	18 (24.3%)	36 (24.3%)	
	Poor informed consent process carried out	2 (2.7%)	4 (2.4%)	
	Privacy not protected	3 (4.1%)	14 (9.5%)

At the individual category level, we found that in the domain of management problems, there were significantly fewer problems identified with the institutional processes among the ICU complaints (*p* < 0.001), whereas in the domain of relationship problems, the ICU complaints were more related to communication and less to listening (*p* = 0.002). At the type level, the five most common types of problem raised by the complaints to the ICUs were those related to facility and equipment functions (76 problems), uncomfortable physical surroundings (68 problems), poor manner of the staff (50 problems), acknowledged problems not responded to (48 problems), and patient handling (31 problems) (Table 2).

Based on the classification of the HCAT, Table 3 shows the severity of problems, their related stages of care, levels of patient harm, and staff groups to which the complaints referred. Although only small proportions of complaints were related to operations and procedures in both settings, the ICU complaints were less related to this stage of care than the general wards (3.9% vs. 6.6%, respectively, *p* = 0.044). Although low severity accounted for more than half of the problems identified in both groups, the ICU complaints had significantly more of medium and high severity than ward complaints (48.3% vs. 37.6%, *p* < 0.001). Problems raised from the ICU complaints were also related more to the care stage of “care on the ward” (indicating care in the ICU referring to the ICU complaints) (*p* < 0.001), and related to a higher proportion of doctors and nurses, summing up to nearly 50%, as the staff group to which the complaints referred (*p* < 0.001). Nevertheless, these two groups of complaints identified similar degrees of patient harm, with “no harm” accounting for more than 85% in both groups (*p* = 0.162). Of the 1029 complaints, those of medium and high severity accounted for 103 (47.7%) of the 216 complaints via phone call, 33.2% (202/608) of those via feedback sheets, 57.9% (55/95) of those by written complaint letter/mail/fax, and 50.6% (44/87) of those provided in person. The written complaints more frequently addressed problems of medium to high severity compared to phone calls (57.9% vs 33.2%, *p* < 0.001).

**Table 3:** Severity, outcomes and referrals of healthcare complaints related to intensive care units and the general wards

Variable	Number of problems, ICUs (*n* = 441)	Number of problems, general wards (*n* = 818)	*p* value
Severity			
Low	228 (51.7%)	510 (62.3%)	< 0.001
Medium	183 (41.5%)	248 (30.3%)	
High	30 (6.8%)	60 (7.3%)	
Stage of care			
Admission	8 (1.8%)	74 (9.0%)	< 0.001
Examination and diagnosis	7 (1.6%)	51 (6.2%)	
Care on the ward	267 (60.5%)	443 (54.2%)	
Operation and procedure	17 (3.9%)	54 (6.6%)	
Discharge	14 (3.2%)	49 (6.0%)	
Unspecified or other	128 (29.0%)	147 (18.0%)	
Level of harm			
N/A	387 (87.8%)	710 (86.8%)	0.162
Minimal	13 (2.9%)	48 (5.9%)	
Minor	19 (4.3%)	25 (3.1%)	
Moderate	10 (2.3%)	21 (2.6%)	
Major	4 (0.9%)	5 (0.6%)	
Catastrophic	8 (1.8%)	9 (1.1%)	
Staff group to which the complaint referred			
Unspecified	161 (36.5%)	277 (33.9%)	< 0.001
Doctor	92 (20.9%)	202 (24.7%)	
Nurse	124 (28.1%)	143 (17.5%)	
Other professional	4 (0.9%)	12 (1.5%)	
Other workers	39 (8.8%)	112 (13.7%)	
Administrative staff	19 (4.3%)	65 (7.9%)	
Other	2 (0.5%)	7 (0.9%)	

N/A: No information on harm is reported

Of the 343 complaints to ICUs, only nine (2.6%) were found to have a coincident report by the healthcare workers as a patient safety event to the institution through the Incident Reporting System. These complaints included one in the “quality”, one in the “safety”, two in the “environment”, two in the “listening”, one in the “communication”, and two in the “respect” categories. Only 10 (1.5%) of the complaints to the general wards were found to have a coincident report as a patient safety incident, including two in the “quality”, two in the “safety”, two in the “environment”, one in the “process”, one in the “listening”, one in the “communication”, and one in the “respect” categories. Overall, only 1.8% of the complaints in this study had a coincident patient safety event reported.

Although the contents of the complaints were highly diverse, examples from each category are provided below to illustrate the concerns from the ICU patients and their family members (translated from the original Chinese text):

“… My mother’s blood vessels were very difficult for the doctors to put a catheter in. A doctor tried many times but all attempts failed. No one helped this doctor, and my mother suffered greatly. Would this cause worsening of her disease? …” (“quality” category)

“… We noticed water accumulating in the breathing tubes connected to the mechanical ventilator, and we heard some beeping noises coming from the ventilator. We were worried that the water might go into our father’s lungs, and talked to the nurses and the doctor, but they were so busy that we were not sure if they had noticed our concerns …” (“safety” category)

“… The ICU was too busy and the healthcare workers looked after too many patients and there was not even enough equipment such as a percussion machine. The patient had to wait until the equipment had been borrowed from another ICU. We think that this delayed therapy and even the recovery of the patient, and it is definitely a serious concern …” (“environment” category)

“We had great expectations about the rehabilitation program for our father, and we were informed that the doctor and therapists would come to visit and provide rehabilitation as scheduled. We felt very upset that there was often a delay in starting this program, and that this did not help my father to recover from his serious illness …” (“institutional process” category)

“We were very concerned about the test results after father received dialysis, but the doctor told us to listen to him about his explanation of father’s condition, and we had no time to ask about this question. We were not sure that the doctor understood our concerns …” (“listening” category)

“… The doctors told us that father would be transferred to the general ward, but we had not been informed in the past several days. We thought his condition had not improved, but they said his vital signs were stable …” (“communication” category)

“The manner of the nurse was terrifying so that the patient was scared. We were uncomfortable about the way the nurse talked to the patient because we saw this as disrespect …” (“respect and patient rights” category)

### Multivariate analyses of factors associated with the presence of problems of medium and high severity

Table 4 shows the results of multivariate logistic regression analyses of factors associated with the presence of problems of medium and high severity identified in the complaints. We analyzed the factors based on two sets of data. In the first analysis based on the 1029 complaints issued, we found that complaints to the ICUs (odds ratio = 1.769, *p* = 0.001), relationship as the main problem domain (OR = 1.523, *p* = 0.007), the recognition of patient harms (OR = 6.100, *p* < 0.001), doctors as the main staff group to which the complaint referred (OR = 2.637, *p* < 0.001), and female complainer (OR = 1.148, *p* = 0.027) were associated with an increased probability of the main problems being of medium and high severity, whereas a complaint issued via feedback sheets (OR = 0.717, *p* < 0.001) was associated with a decreased probability of the main problems being of medium and high severity.

**Table 4:** Multivariate logistic regression analyses of variables associated with problem of medium or high severity raised in the complaints

Variable	Odds ratio	95% confidence interval	*P* value
Analysis 1 (based on the 1029 complaints)			
Complaint to the ICUs	1.769	1.260–2.484	0.001
Relationship as the main problem domain	1.523	1.121–2.069	0.007
“Care on the ward” as the main stage of care	1.203	0.909–1.592	0.197
Any harm	6.100	3.602–10.332	< 0.001
Doctor as the main staff group referred to	2.637	1.837–3.787	< 0.001
Complaint issued by family member or surrogate	1.036	0.739–1.453	0.338
Female complainer	1.148	0.865–1.525	0.027
Complaint issued via feedback sheet	0.717	0.533–0.964	< 0.001
Analysis 2 (based on the 1259 problems)			
Complaint to the ICUs	1.838	1.361–2.482	< 0.001
Relationship as the problem domain	1.608	1.230–2.102	0.001
“Care on the ward” as the stage of care	1.220	0.946–1.572	0.125
Any harm	6.295	4.008–9.887	< 0.001
Doctor as the staff group referred to	2.150	1.565–2.955	< 0.001
Complaint issued by family member or surrogate	0.977	0.720–1.326	0.882
Female complainer	1.288	1.001–1.659	0.049
Complaint issued via feedback sheet	0.726	0.556–0.947	0.018

In the second analysis based on the 1259 problems identified in the complaints, we found that complaints to the ICUs (OR = 1.838, *p* < 0.001), relationship as the main problem domain (OR = 1.608, *p* = 0.001), the recognition of patient harms (OR = 6.295, *p* < 0.001), doctors as the main staff group the complaint referred to (OR = 2.150, *p* < 0.001), and female complainer (OR = 1.288, *p* = 0.049) were associated with an increased probability of problems of medium and high severity, whereas a complaint issued via feedback sheets (OR = 0.726, *p* = 0.018) was associated with a decreased probability of problems of medium and high severity.

## Discussion

In this study, we estimated the rate and density of healthcare complaints to the ICUs. The complaint rate in the ICU was lower than the overall complaint rate for the inpatient setting in this hospital. We found that most of the complaints to the ICUs were issued from the family and surrogates, focused on the environmental issues when the problems were in the management domain and involved more problems focused on communication when the problems were in the relationship domain. Complaints to the ICUs were independently significantly associated with problems of higher severity among all complaints in the inpatient care setting. These findings provided insights into the opportunities for improving the care of patients in the ICU.

In addition, this study shows that the complaints provided information that may have been difficult to obtain from other sources such as the institutional Incident Reporting System. In this study, only 1.8% of the complaints had a coincident report as a patient safety event, indicating that the institution may not have received, at least in a timely manner, important information required to improve the quality of healthcare and services for the patients. The predominant categories in each domain of problems, such as quality versus safety, environment versus process, and listening/respect versus communication, suggest that complaints may be valuable as a complimentary tool to help understand the whole picture of healthcare and service quality provided by the institution.

Overall, the complaint rate related to the inpatient setting in our hospital was not as low reported in the literature [19]. A possible explanation is that our hospital has established multiple channels for the patients and their families to provide their complaints, which are all formally managed and filed by dedicated workers. In a study during 1997 to 2001, Taylor et al. demonstrated similar complaint rates in general wards and ICUs [19]; our study showed a lower rate of complaints to the ICUs than that to the general wards. Since we were unable to investigate the complaints before the year 2008, we did not know if there was a trend toward an increased complaint rate in the general wards than in the ICUs after 2001. However, the patients and families at our hospital applied multiple channels to issue complaints, like the feedback sheets, in addition to the traditional complaint process. It might be difficult to distinguish between comments with negative feedback and complaints, and other informal comments such as telephone calls, threats of lawsuits, and comments to caregivers commonly provided to the hospital as the sources for the collection of patient feedback [21]. The finding that the family and surrogates accounted for most of the contacts of the complaint is compatible with the premise that patients treated in the ICU setting might have difficulty in direct expression of undesirable experience in the care they received.

Our data may also reflect the healthcare context in Taiwan for general ward patients, in that there is almost no limitation on visits to the patient by family members or friends during the night shift, which is in contrast to the strict regulations for visiting patients in the ICU. The family members of the patients in the ICUs, with limited direct observation of the ongoing care and the inability of patients to express their needs and experience, may have had a quite different understanding of care compared to the general ward setting. We suggest that the differences in care setting and background context mean that it is necessary to understand the problems and concerns of care quality for the patients staying in an ICU.

Our finding that the patients made very few of the complaints that were issued suggests the need for a more effective channel to understand the unsatisfactory experience according to the ICU patients. Nevertheless, opinions from the family members might provide useful information on the healthcare system, as satisfaction among the families of critically ill patients has attracted increased concern as a primary indicator to evaluate care quality in the ICU. Previous reports about the surveys of ICU patients were mostly performed after surviving patients had left the ICU, but this could miss a significant proportion of non-survivors of the ICU. Survey of family satisfaction using a validated questionnaire has been suggested to assess satisfaction in such settings [2], but this type of survey might not render all of the answers to questions about the understanding of the experience by the patients and families. These tools might explore helpful information but usually request the responders to rate the experience under the pre-determined dimensions [22]. In a literature review of studies evaluating family satisfaction in the ICU, the mean response rate was 65.5%, and response rates greater than 70% were found only in 28.2% of the studies. Investigation of the complaints might, therefore, provide complementary understanding in this setting [12, 23]. As there was limited reporting of a structured analysis of complaints to the intensive care setting, we suggest that the hospitals should stress the analysis of healthcare complaints in this setting. Analyzing healthcare complaints systematically and recognizing situations and providers likely to generate complaints are critical steps in designing strategies for reducing complaints [23].

Patient safety was one of the main concerns raised by the patients and their family members through complaints and by the healthcare workers through the institutional incident reporting system. Nevertheless, the issues of safety raised by the complainers may be attributed to their understanding of patient safety. In addition, the complainer may have raised concerns related to conflicts about their expectations about care quality and the care environment. The classification of the complaints was derived using the HCAT; however, this does not mean that the patients were not at any risk of harm when there were complaints about environment and relationship issues. A broader definition of patient safety may include the additional risk of patient harm unrelated to the disease and treatment, and thus the issue of patient safety may be more wide-reaching than the “safety” category alone.

Our use of the structured classification and analysis of complaints to the intensive care setting as suggested by the HCAT may provide valuable insights into the clinical, managerial, and relationship problem raised by complaints in the intensive care setting. That the ICU complaints focused more on the environment than the institutional processes when the problem was in the management domain reflects the perception of the care environment, which might be very important for the family and surrogates. Also, the ICU complaints had more issues in the communication category than the ward complaints; this suggests that the complexity of care in the ICU might need better communication when the family faces the patient having a severe clinical condition and has important decisions to make. Indeed, the ICU environment was shown to be related to family satisfaction, which is reported to possibly be increased by 6% in the new ICU environment with noise-reduced, single rooms with daylight, adapted coloring and improved family facilities [24]. The report by Heyland et al. shows that the family is least satisfied with the waiting room atmosphere and frequency of physician communication [25]. Other themes for improvement reported in the literature included ICU atmosphere, amenities for visiting relatives, emotional support, consistency, clarity, and completeness of information, respect and compassion towards families, the inclusion of family, and support during decision-making processes [3, 4, 6, 25]. Despite the fact that family and relatives of the patients in ICU are expected to have both a receiving and participating role [26], families might also encounter unsupportive interactions, which might result from poor communication [27]. Our study findings provided further evidence of these observations, and stress the need to facilitate communication and interaction to relieve the anxiety and distress experienced by the families of the critically ill [27].

The use of patient satisfaction data to improve the process of care in the ICU has been recommended [28, 29]. As awareness and knowledge about healthcare has increased, demand from the public and individuals has surged. Likewise, complaints from patients could serve as a management tool for continuous quality improvement [17]. Therefore, healthcare should incorporate patient feedback as an integral part of quality improvement [17]; the patient’s perception of the quality of health services might come from the complaints [30]. Indeed, patients and their families might identify many issues as shown in our study that the existing healthcare quality frameworks do not cover [31]. While responsiveness remains a major domain of health system performance [32], the use of complaints to enhance the responsiveness might provide timely action for quality improvement. Despite difficulties remaining in the implementation of strategies to improve family satisfaction [33], we believe the analysis of healthcare complaints, including those to the ICUs, is of high value in quality improvement.

There are limitations to this study. First, this was a retrospective analysis based on the recorded opinions provided by the complainers, while the detailed information related to the problems during the interaction performed between the staff members of the complaint management and the complainers were not available; therefore, we cannot verify the problems, especially with the healthcare workers. Second, the original platforms for complaining and data entry of the complaint contents were not structurally designed to align with the HCAT; therefore, the study might miss some relevant issues as ideally recommended by HCAT. Nevertheless, as a significant proportion of complaints are still provided by the channels without direct contact or direct communication, the staff members of the complaint management or patient relations might not have the chance to go into details of the experience reported by the complainers. Third, some of the complaints were issued anonymously, therefore, we were unable to collect more detailed administrative data related to patient care. Indeed, there might be a trade-off process to obtain more opinions within the setting of anonymity, but with the loss of specificity on the understanding of the institutional care process and management issues. Fourth, the relatively small number of some types of problems found in the complaints might now be equivalent to the degree of patient satisfaction. We believe that the scarcity of healthcare complaints does not necessarily determine the fulfillment of high quality or complete satisfaction. Complaint management requires engaged personnel to understand the opinions of the complainers, similar to qualitative methods in the healthcare studies, especially in acute medicine such as in the ICU setting [34, 35]. To obtain more details and rationales for improvement, future prospective study of the combination of complaint interview and qualitative analysis is warranted.

## Conclusions

In conclusion, complaints to the ICUs were associated with a higher severity of problems raised by the complaints to the institution. The analysis of healthcare complaints may provide valuable insights into the experience of care that the patients perceived in the intensive care setting, those usually issued from the family members of the patients, who might need more satisfactory communications and appreciation of a more comfortable care environment. The Healthcare Complaints Analysis Tool may render useful structure to classify and understand complaints in the intensive care setting, and thus warrants the establishment of this system for prospective collection of complaints data for the healthcare system.

## Additional file

Additional file 1: The process of healthcare complaint analysis using HCAT (Healthcare Complaints Analysis Tool). (PDF 77 kb)

## Abbreviations

HCAT: Healthcare Complaint Analysis Tool
ICU: Intensive care unit
