# Supplementary material for: Comparison of complaints to the intensive care units and those to the general wards: an analysis using the Healthcare Complaint Analysis Tool in an academic medical center in Taiwan
Source: Crit Care. 2018 Dec 6;22:335. doi: 10.1186/s13054-018-2271-y (PMC6282256; doi:10.1186/s13054-018-2271-y)
Supplement: Supplementary file 1 — The process of healthcare complaint analysis using HCAT (Healthcare Complaints Analysis Tool). (PDF 77 kb) [file 13054_2018_2271_MOESM1_ESM.pdf]

Supplementary File

Comparison of complaints to the intensive care units and those to the general wards: An analysis using the Healthcare Complaint Analysis Tool in an academic medical center in Taiwan

Jih-Shuin Jerng, Szu-Fen Huang, Hsin-Yu Yu, Yi-Chun Chan, Huang-Ju Liang, Huey-Wen Liang, Jui-Sheng Sun

The process of healthcare complaint analysis using HCAT (Healthcare Complaints Analysis Tool)

To obtain a quantitative description, the investigators coded each complaint according to the taxonomy of the Healthcare Complaints Analysis Tool (HCAT) [1]. HCAT, which was developed by Gillespie and Reader and is free for practitioners and researchers to use, is intended to be a standardized tool for analyzing healthcare complains, which provides assessment of problem severity with reliability [1]. HCAT provide guidelines for coding healthcare complaints using HCAT, including the coding based on empirically identifiable text, without judging intention and importance, rating the highest severity independently of outcome (i.e., harm), association with stage of care, and relating harm exclusively to the incident complained about [1]. The investigators, including the coders, adopted the process recommended by this paper for a structured classification of complaints (see Supplementary Data of Reference 1).

Before the analysis, all of the records of complaint cases to be analyzed were retrieved from the database of the Center for Quality Management of this institution. The original records were obtained from a variety of channels, including written and non-written information, and transcribed into electronic text files. Each case was formally identified as a healthcare complaint and was given a serial identification number.

For the analysis, the coder first identified the problems and assessed the severity of each complaint issued during the study period. The coder independently analyzed the complaints by reviewing the contents of the text describing the complaints to see if the complaints were related to at least one of the three domains, including clinical, management, and relationship, and subdivided them into seven categories. The clinical domain pertained to the quality of care and patient safety; the management domain referred to environmental management and institutional processes related to the handling of patients; and the relationship domain included communication, listening and respect/patient rights [1]. The coder then determined the type (sub-category) of the problem found as suggested by HCAT. Further, the severity of each problem category found was determined according to HCAT; only the highest level of severity was recorded in one identified category of problem. Multiple categories were allowed, but only one level of severity was allowed for each

identified category. The coder evaluated the complaints in a non-judgmental way of the complainers, patients or healthcare workers.

In the next step, the coder then specified the stages of care to which the complaints were related, including admissions, examinations and diagnoses, care on the ward, operation/procedures, discharge/transfers, and unspecified or other [1]. The coder then determined the level of harm reported and considered as overall harm caused to the patient by the problem raised by the complaint, classified as no information on harm, minor harm, moderate harm, major harm, and catastrophic harm, by identifying related texts from the filed case contents indicating the severity.

The coders then together reached a consensus for each case about the final coding results after discussing the available records related to the complaints. Regardless of the original channel of the complaints, the coding of all of the complaints and problems in this study were exclusively based on the text records from the file database from the Patient Relations Office of this institution, while the complaint severity was assessed according to the contents of the text without any additional interviews with the staff members who originally managed the cases or the healthcare workers caring for the patients.

After the above classification had been completed, the coder then summarized the descriptive data in detail, such as the demographics of the complainers, and the distribution of staffing groups being complained about.

References

1. Gillespie A, Reader TW. The Healthcare Complaints Analysis Tool: development and reliability testing of a method for service monitoring and organisational learning. BMJ Quality & Safety 2016,25:937-46.
